# Piecewise-potential-field-based path planning method for fixed-wing UAV formation

**DOI:** 10.1038/s41598-023-28087-0

**Published:** 2023-02-08

**Authors:** Yuxuan Fang, Yiping Yao, Feng Zhu, Kai Chen

**Affiliations:** College of Systems Engineering, National University of Defense Technology, Changsha, 410073 China

**Keywords:** Aerospace engineering, Computational science, Software

## Abstract

The multi-UAV path planning method based on artificial potential field (APF) has the advantage of rapid processing speed and the ability to deal with dynamic obstacles, though some problems remain—such as a lack of consideration of the initial heading constraint of the UAVs, making it easy to fall into a local minimum trap, and the path not being sufficiently smooth. Consequently, a fixed-wing UAV formation path planning method based on piecewise potential field (PPF) is proposed, where the problem of UAV formation flight path planning in different states can be solved by suitable design of the PPF function. Firstly, the potential field vector can be used to represent the potential field functions of obstacles and target points to meet the kinematic constraints of the UAV. Secondly, the local minimum region can be detected, the additional potential field vector being set to break away from this region. Finally, the change rules of the potential field vector of a UAV in the formation reconstruction scene can be designed, a smooth formation flight track being assured by adjusting the corresponding speed of each UAV track point. Considering the path planning of a five-UAV formation as an example, we conducted simulation experiments. The results showed that—compared with the existing methods based on APF—the results obtained using the PPF-based method considered the initial heading limits of the UAVs, the planned path being considerably smoother. Moreover, the proposed method could plan multiple UAV tracks, satisfying the known constraints without conflict in complex scenarios.

## Introduction

At present, unmanned aerial vehicle (UAV) are widely used in many fields, including disaster detection, low-altitude reconnaissance, atmospheric research, communications, disaster area search, and rescue^1–3^. These can involve tasks that pose security risks or require long periods of continuous operation, making them unsuitable for manned aircraft. But UAV is most suitable for such missions^4^. Compared with rotor-based UAV, fixed-wing UAV has many advantages, including long flight distances, long flight times, high speeds, and heavy loads. They are suitable for missions with long, continuous working hours and stringent requirements for airborne equipment^5^. Currently, the mission capability of a single UAV can be limiting, so a UAV formation could improve the efficiency of the mission^6^. A reasonable formation can reduce task costs (such as saving fuel) and improve the effectiveness of a mission (such as increasing the search scope)^7^. The planning of a smooth, feasible route for each UAV based on kinematic constraints and preset formation requirements—which can reduce the difficulty of UAV trajectory tracking—is an important task in current fixed-wing UAV formation research^8,9^. Consequently, in this paper, the flight path planning of fixed-wing UAV formation is studied.

The flight path planning of a UAV requires a path for the UAV from its starting point to the end in a space where there are obstacles and other restricted areas^10,11^. Fixed-wing aircraft cannot take off and land vertically or fly sideways and they have a minimum flight speed limit, characteristics which require that the planned route satisfies its constraints. At present, the most commonly used method for UAV path planning is the global search algorithm, such algorithms using grid graphs^12^, Voronoi graphs^13^, and other theories to divide up the search space. In this way, the planning space is discretized, making the flight path more tortuous. Consequently, it becomes necessary to smooth the path^14^ using geometric methods^15^—such as the B-spline and Bezier curves, or the Euler spiral^16^—or obtain a relatively smooth path directly by thinning the partition space and adding constraints. However, increasing the computational overhead to accommodate an increase in the degree of refinement considerably reduces the efficiency of the algorithms, resulting in these algorithms losing their performance advantage^17,18^.

When the problem is extended from single UAV path planning to cooperative UAV-formation path planning, the additional constraints that need to be considered limit the planning methods based on the global search algorithm enormously, as these algorithms have difficulty coping with dynamic mission scenarios. Although it can be difficult to maintain the most favorable flight path obtained using online search algorithms, they can effectively deal with UAV formation planning in the context of dynamically changing targets and obstacles^19^. Among them, the artificial potential field (APF) method obtains the track by simulating the attraction from a target and the repulsive force from an obstacle, so that it can plan the track in a three-dimensional space, maintain good performance, and deal with dynamic targets and obstacles^20–22^. However, UAV formation path planning methods based on APF have several problems—including the track easily falling into local minimum areas, the planning result not being sufficiently smooth^23^ and do not consider the starting flight direction of the UAVs. Moreover, it can be difficult to meet the path requirements of the UAVs, as many methods lack the coordination rules among members of the UAV formation, leading to the great independence between friendly aircraft and a lack of formation characteristics.

Consequently, a fixed-wing UAV formation path planning method based on piecewise potential field (PPF) is proposed in this paper. The main contributions of this paper are as follows:

1. A UAV model is established to provide kinematic constraints for track planning based on the motion characteristics of fixed-wing aircraft. A UAV formation model is established based on the “lead-plane–wingman” structure, so the coordination rules of the formation members can be determined
2. The potential field function is designed based on the state of different stages of the UAV formation flight. Firstly, the potential field function from an obstacle and target point can be expressed by a potential field vector, and searched based on the UAV model, so that the obtained path meets the kinematic constraints and obstacle avoidance requirements of the UAV. Secondly, the direction of motion of the UAV can be changed by detecting a special position where the potential field vector from the obstacle is parallel to the flight direction of the UAV, and setting an additional potential field vector to be perpendicular to the flight direction of the UAV to avoid the planning results falling into a local minimum area. Finally, the dynamic change rules of the potential field vector of teammates in the formation reconstruction scene can be recommended to meet the smoothing requirements of the flight path planning results by adjusting the speed of the UAV rendezvous.

It should be noted that the scenario addressed in this study is at the UAV path planning level, which does not involve the design of the UAV flight control system itself^5^.

This paper is organized as follows: firstly, we introduce current related research. Secondly, we introduce the models of a UAV and UAV formation. Thirdly, we introduce the route planning method proposed in this paper. Fourthly, we presents the experimental results and analyses. Finally, we presents the conclusions and future work.

## Related work

### Research on path planning method of single UAV

The path planning of a single UAV has always been a research hotspot. Jothi and Akshya solved the coverage path planning problem and proposed a deep learning framework based on a graph convolution network, which could be used to find the optimal route of a traveling salesperson problem^24^. However, this study focused on the level of task planning rather than on the specific details of the path.

- Research on single UAV path planning considering computational efficiency

The problem of UAV path planning becomes progressively more complex when considering the actual environment and dynamic constraints of UAVs, which can be challenging for global search algorithms—including sampling-based algorithms represented by the A* algorithm, and intelligent algorithms^25^ represented by the genetic algorithm^26^, ant colony optimization (ACO) algorithm^27–29^, particle swarm optimization (PSO)^30^, and hybrid algorithms^31,32^. Jamshidi et al. used the parallel improved grey wolf optimization algorithm to overcome this complexity and obtain results relatively quickly^33^. Liu et al. improved the basic whale optimization algorithm (WOA), the global search ability and convergence speed of the improved WOA being better than those of the WOA, artificial bee colony, and PSO^25^. Zhou et al. imitated the characteristics of plant phototaxis and proposed a plant growth algorithm to quickly generate paths in space with obstacles^34^. These studies improved the efficiency of the algorithms but did not optimize the path itself to adapt to the motion characteristics of fixed-wing UAVs.

(b) Research on single UAV path planning for optimizing smoothness

Some scholars have studied the smoothness requirements of fixed-wing UAV path-planning results. Tian et al. improved the elastic rope algorithm to solve the problem of the algorithm being prone to second-order discontinuity of the path—that is, a non-smooth path^14^. Phung and Ha proposed spherical vector-based PSO based on the search space, which focused on the security and feasibility of generating routes. This algorithm could generate a smooth path but had several altitude constraints, which were not conducive to the flight of fixed-wing UAVs^17^. These studies improved the algorithms to obtain a smooth path but still did not consider the initial orientation of the UAV.

(c) Research on single UAV path planning considering dynamic environment

At the same time, these algorithms only considered fixed obstacles and targets in the planning process, having considerable limitations in multi-UAV formation scenes where targets and obstacles were constantly changing. At present, online path-planning algorithms such as the dynamic window approach (DWA), rapid exploring random tree (RRT)^2^, and APF^35,36^ methods are more suitable for dealing with dynamic environments. Naderi et al. improved the RRT algorithm and realized the path search of the RRT method under real-time conditions. However, this method was not suitable for large-scale planning and borderless space—such as the three-dimensional space of UAV flights^13^. Wang et al. used Kalman filtering to predict the position of a target and the ACO algorithm to optimize the tracking path of multiple targets, improving the tracking ability of a UAV to multiple dynamic targets. However, the algorithm did not study scenes with obstacles^37^. A dynamic path planning algorithm based on obstacles’ position prediction and modified APF—HOAP proposed by Feng et al., could effectively deal with the influence of dynamic obstacles. However, the algorithm did not consider the motion characteristics of fixed-wing UAVs, its planning results permitting UAVs to avoid obstacles by hovering first before making a detour^19^. Bai et al. gridded the obstacles and then combined the DWA algorithm with the improved A* algorithm to plan shorter and smoother paths and deal with dynamic obstacles^38^. However, the algorithm was only verified in a two-dimensional space and had higher computational requirements for grid processing.

### Research on cooperative flight path planning of multiple UAVs

To realize the formation flight of a fixed-wing UAV, in addition to the path planning of a single UAV, a cooperation strategy among multiple UAVs also needed to be studied. Wei and Xu improved the construction method of the Voronoi diagram and combined it with the dual decomposition ACO algorithm to realize the path planning of multirotor UAVs^1^. However, the UAVs suitable for this method operated independently of each other, and there were no cooperative flight formation characteristics.

- Research on multi-UAV path planning for formation flight

Muslimov and Munasypov proposed an approach based on Lyapunov nonuniform functions in both the magnitude and direction path-following vector fields to coordinate a formation of fixed-wing UAVs by decentralized consensus^39^. However, the patrol formation in this study was designed in advance, and the algorithm focused on the bottom control of the UAV. Consequently, the formation was limited to a circular formation and could not be specified based on the needs of a mission. Manathara and Ghose designed specific motion control rules in combination with Dubins curves and realized a multi-aircraft cooperation algorithm in which a multi-UAV formation adjusted its motion state using the estimated time of arrival parameter to ensure that it could reach a rendezvous point at the required time and considered the avoidance of motion obstacles^40^. However, the scene designed for this study was simple, and the UAV kinematic model was simplified with no formation coordination between the UAVs.

(b) Research on multi-UAV path planning based on APF

At present, many scholars improve the APF method to realize the formation path planning of multi-UAV. A detailed helicopter dynamics model was designed by Paul et al., and the formation reconstruction and obstacle avoidance were realized based on extended local potential field based on a virtual leader, but there is no specific description of the local minimum processing method, and the kinematic characteristics of the helicopter and fixed-wing aircraft are also very different^41^. By establishing the bounded mapping relationship between the kinematic model parameters and the potential field values of the fixed-wing UAV, the trajectory that meets the requirements of kinematic constraints and obstacle avoidance can be generated. Among them, Feng et al. used polar coordinates to describe the formation to ensure that the UAV can form a closer formation when it encounters obstacles, but they have not studied the possible local minimum problem^42^, which was solved by Zhao et al. through using small disturbances^43^. However, both of them are only studied in two-dimensional space. Chen et al. used an APF to realize UAV-formation flight, but the flight path of the UAV oscillates greatly when avoiding obstacles. Consequently, this method could not be fully applied to fixed-wing UAVs^23^. Zhang et al. designed a consensus control algorithm and combined with the APF method and obstacle avoidance rules to realize path planning in three-dimensional space, but did not analyze the feasibility of track in the aspect of kinematics and the problem that the planning method is easy to fall into local minima value^44^. However, in the follow-up research, they solved these problems to some extent by designing appropriate UAV models and using rotation potential field vectors to avoid collision, but the proposed planning method was only suitable for the specific formation of three UAVs and the experimental scene is too simple, so the formation adjustment was more complex and it is difficult to prove the universal applicability of this method^45^. The latest research of Liu et al. can avoid the local minimum problem and the local oscillation of trajectory by constructing a novel APF function with anisotropy in each dimension. This study also proposed a target vector decomposition strategy to avoid dynamic obstacles and sudden threats in advance, so that the planned trajectory does not have a segment with sharp changes in normal acceleration, but this study focuses on the cooperation of multiple aircraft where the problem of formation flight of multiple UAVs has not been studied^46^.

In summary, there are many problems in the current related research—including unsmooth paths, no consideration of the initial UAV orientation, no consideration of the dynamic environment, and the lack of a formation cooperative flight strategy among multiple UAVs. At the same time, the improved method based on APF also has some limitations in the application scenario of fixed-wing UAV formation flight. Consequently, this paper proposes a fixed-wing UAV formation path planning method based on the PPF to realize the formation flight planning of multi-UAVs in a preset scene.

### Theory: fixed-wing UAV and formation modeling

This section introduces the kinematic model and formation cooperative model of fixed-wing UAV used in this paper^47^. In the UAV kinematic model, the linear velocity along the flight direction of the UAV and the angular turning velocity are set, and the linear acceleration is set in the model too. In the formation cooperative model, the avoidance priority among friendly aircraft is set based on the "Lead-plane-Wingman" formation control structure, and the wingman target point is dynamically calculated according to the target formation and the position of lead plane.

### Fixed-wing UAV model using direction restriction

**Figure 1:**
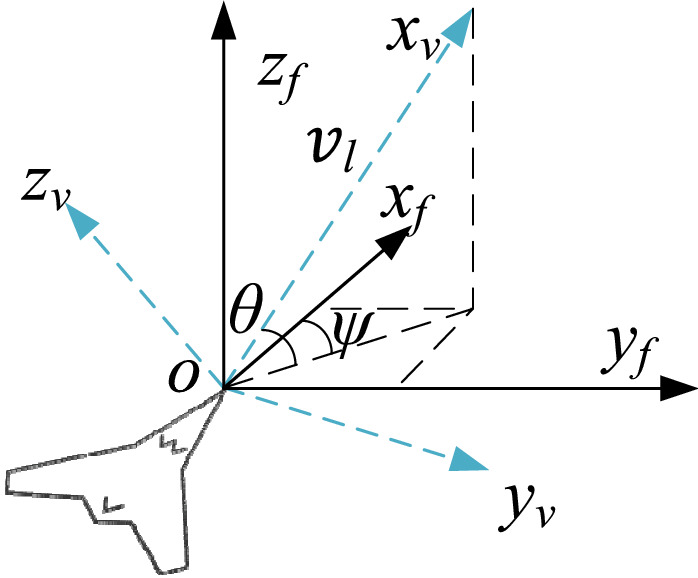
UAV kinematics model.

Figure 1 defines the inertial coordinate system (ICS)—that is, the coordinate system represented by the black solid line in the figure—and the vehicle coordinate system (VCS)—that is, the coordinate system represented by the blue dotted line in the figure—to describe the state of a UAV in the planning space and from the perspective of the UAV, respectively. Based on the motion characteristics of fixed-wing aircraft, the position state matrix ***P*** of a fixed-wing UAV in the planning space can be defined as follows:

1 $$\begin{array}{c}{{\varvec{P}}=\left[\begin{array}{ccccc}x& y& z& \theta & \psi \end{array}\right]}^{T},\end{array}$$

where *x*, *y*, and *z* are the coordinate positions of a UAV in the ICS and θ and ψ represent the attitude information of the UAV. θ is the pitch angle (− π/2 ≤ θ ≤ π/2), and ψ is the yaw angle (azimuth) (− π ≤ ψ ≤ π).

The motion state of a UAV can be described by the velocity matrix ***V*** and acceleration matrix ***a,*** as follows:

2 $$\begin{array}{l}\left\{\begin{array}{c}V={\left[\begin{array}{lll}{v}_{l}& {\omega }_{\theta }& {\omega }_{\psi }\end{array}\right]}^{T}\\ a={\left[\begin{array}{lll}{a}_{l}& {\alpha }_{\theta }& {\alpha }_{\psi }\end{array}\right]}^{T},\end{array}\right.\end{array}$$

where the linear, pitching angular, and yaw angular velocities of the UAV are denoted by ${v}_{l}$, ${\omega }_{\theta }$, and ${\omega }_{\psi }$, respectively. The linear, pitch angular, and yaw angular accelerations of the UAV are denoted by ${a}_{l}$, ${\alpha }_{\theta }$, and ${\alpha }_{\psi }$, respectively.

The following assumptions can be made regarding the kinematic characteristics of a fixed-wing UAV:

1. The linear acceleration of the UAV is constant—that is, ${a}_{l}$ is constant. However, the values for acceleration and deceleration can differ.
2. The UAV turns at a constant angular velocity—that is, ${\alpha }_{\theta }$ and ${\alpha }_{\psi }$ are constant. Moreover, the time consumed by the process of changing the flight direction of the UAV can be ignored.
3. The velocities of the UAV have an upper limit, and the linear velocity has a lower limit (${v}_{{l}_{min}}$), which is greater than 0.

Based on the previous hypothesis, the relationship between the velocity and acceleration can be expressed as follows:

3 $$\begin{array}{c}V\left(t\right)=I\cdot V\left({t}^{^{\prime}}\right)+f\left({t}^{^{\prime}}\right)\cdot \Delta t\cdot I\cdot a,\end{array}$$

4 $$\begin{array}{c}f=diag\left({f}_{l},{ f}_{\theta },{ f}_{\psi }\right),\end{array}$$

where *I* is the unit matrix; $\Delta t(\mathrm{s})$ is the step size of the time advance, $t$ = ${t}^{\mathrm{^{\prime}}}+\Delta t$; and ${\varvec{f}}$ is the velocity change trend matrix of the UAV, the value of which will be described in the next section.

Consequently, the relationship between the position and attitude of the UAV in the planning space as well as its velocity can be obtained as follows:

5 $$\begin{array}{l}\left\{\begin{array}{c}\dot{x}={v}_{l}\mathrm{cos}\theta \mathrm{cos}\psi \\ \dot{y}={v}_{l}\mathrm{cos}\theta \mathrm{sin}\psi \\ \dot{z}={v}_{l}\mathrm{sin}\theta \\ \dot{\theta }={\omega }_{\theta } \\ \dot{\psi }={\omega }_{\psi }.\end{array}\right.\end{array}$$

### UAV formation model based on “lead-plane–Wingman” structure

In this paper, the coordination rules of formation members can be determined based on the “lead-plane–wingman” formation structure. When flying in formation, each UAV treats its teammates as moving obstacles and avoids them based on a preset priority order. To form a specified formation, this paper sets the target point of the lead plane in advance, before determining the local path target point in the planning process of a wingman based on the path results of the lead plane and the formation requirements^23^. If the lead plane is UAV 0, then the equation for calculating the local target points of the wingmen at a certain time can be expressed as follows:

6 $$\begin{array}{c}{{\varvec{G}}}_{i}={{\varvec{A}}}_{{\varvec{v}}{\varvec{f}}}\cdot {{\varvec{B}}}_{{\varvec{v}}{\varvec{f}}}{\cdot \left[\begin{array}{ccc}{x}_{i}^{gv}& {y}_{i}^{gv}&{z}_{i}^{gv}\end{array}\right]}^{T}+{\left[\begin{array}{ccc}{x}_{0}& {y}_{0}& {z}_{0}\end{array}\right]}^{T},\end{array}$$

7 $$\begin{array}{c}{{\varvec{A}}}_{{\varvec{v}}{\varvec{f}}}=\left[\begin{array}{ccc}\mathrm{cos}{\psi }_{0}& -\mathrm{sin}{\psi }_{0}& 0\\ \mathrm{sin}{\psi }_{0}& \mathrm{cos}{\psi }_{0}& 0\\ 0& 0& 1\end{array}\right],\end{array}$$

8 $$\begin{array}{c}{{\varvec{B}}}_{{\varvec{v}}{\varvec{f}}}=\left[\begin{array}{ccc}\mathrm{cos}{\theta }_{0}& 0& \mathrm{sin}{\theta }_{0}\\ 0& 1& 0\\ \mathrm{sin}{\theta }_{0}& 0& \mathrm{cos}{\theta }_{0}\end{array}\right],\end{array}$$

where matrix $${{\varvec{G}}}_{i}={\left[\begin{array}{ccc}{x}_{i}^{g}& {y}_{i}^{g}& {z}_{i}^{g}\end{array}\right]}^{T}$$ is the local path target point of the *i*th wingman (*i* = 1, 2, …, *n*) in the ICS; ${{\varvec{A}}}_{{\varvec{v}}{\varvec{f}}}$ and ${{\varvec{B}}}_{{\varvec{v}}{\varvec{f}}}$ are matrices that transform coordinates from the VCS to the ICS; ${\psi }_{0}$ and ${\theta }_{0}$ are the yaw and pitch angles of the lead plane in the ICS, respectively; $${\left[\begin{array}{ccc}{x}_{i}^{gv}& {y}_{i}^{gv}& {z}_{i}^{gv}\end{array}\right]}^{T}$$ is the target point of a wingman under the VCS of the lead plane—that is, the position of the target formation relative to the lead plane; and $${\left[\begin{array}{ccc}{x}_{0}& {y}_{0}& {z}_{0}\end{array}\right]}^{T}$$ is the position of the lead plane under the ICS.

### Methodology: path planning method for UAV formation based on PPF

Based on the characteristics of the UAV formation flight and APF method, this paper classifies the formation flight state of the UAV as follows: ${s}^{1}$ is the state of target following and obstacle avoidance; ${s}^{2}$ is the state in which a local extreme point is encountered; and ${s}^{3}$ is the state of establishing the formation. It should be noted that these three states are not independent of each other. The relationship between them can be expressed in sets as: ${s}^{1}\supset {s}^{2}\supset {s}^{3}$.

Based on the above three states and the models established in the previous section, the potential field function can be improved and divided, and the piecewise potential field (${{\varvec{F}}}_{v}$) obtained. The formal representation of ${{\varvec{F}}}_{v}$ under the VCS can be expressed as follows:

9 $$\begin{array}{c}{{\varvec{F}}}_{iv}\left({{\varvec{S}}}_{i}\right)=\left\{\begin{array}{c}{{\varvec{F}}}_{iv}^{1}\left({{\varvec{S}}}_{i}\right),{{\varvec{S}}}_{i}\in {{\varvec{S}}}^{1}\\ {{\varvec{F}}}_{iv}^{2}({{\varvec{S}}}_{i}),{{\varvec{S}}}_{i}\in {{\varvec{S}}}^{2}\\ {{\varvec{F}}}_{iv}^{3}\left({{\varvec{S}}}_{i}\right),{{\varvec{S}}}_{i}\in {{\varvec{S}}}^{3},\end{array}\right.\end{array}$$

where ${{\varvec{S}}}_{i}({{\varvec{P}}}_{i},{{\varvec{V}}}_{i},{s}_{i})$ represents the state of *i*th UAV, including the position state (${{\varvec{P}}}_{i}$), the speed state (${{\varvec{V}}}_{i})$, and the formation flight state (${s}_{i}$) (${s}_{i}\in \left\{{s}^{1}{,s}^{2},{s}^{3}\right\}$).

### Target following and obstacle avoidance strategy based on virtual vector

In this paper, the virtual vector is used to represent the potential field function in the APF method, the solution being based on the UAV model described in “Fixed-wing UAV model using direction restriction”, so that the planning results meet the motion characteristics of the UAV itself. In the planning space, the UAV receives the attraction vector from a target point and the repulsion vector from an obstacle. As a result, the potential field vector of a UAV in the ${s}^{1}$ state—that is, ${\varvec{S}}\in {{\varvec{S}}}^{1}$—can be obtained.

The target attracts the UAV throughout the planning space. For obstacles, the UAV takes avoidance measures only at the minimum safe distance, which is called the influence radius (*R*) of the obstacle on the UAV—that is, the distance between the UAV and the obstacle when it begins to take obstacle avoidance measures. In this paper, a single obstacle is set as a sphere of radius (${R}_{o}$). The UAV cannot enter the sphere, else it would be regarded as a collision between the UAV and the obstacle.

**Figure 2:**
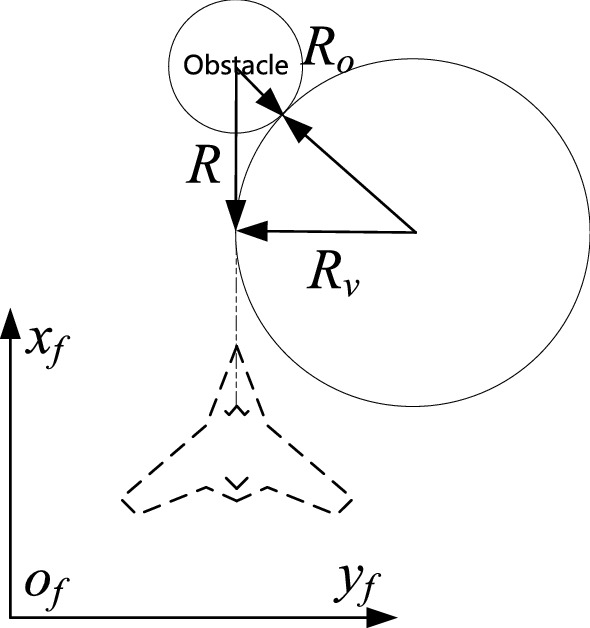
Schematic diagram of UAV obstacle avoidance.

Consider the obstacle distribution scenario shown in Fig. 2, where the obstacle is directly in front of the UAV. The formula for *R* can be expressed as follows:

10 $$\begin{array}{c}R=\sqrt{{\left({R}_{o}+{R}_{v}\right)}^{2}-{R}_{o}^{2}},\end{array}$$

11 $$\begin{array}{c}{R}_{v}={v}_{l}/\omega +{r}_{v},\end{array}$$

where $\omega$ is the maximum angular velocity that the UAV can achieve ($\omega =\mathrm{max}\left(|{\omega }_{\theta max}|,{|\omega }_{\psi max}|\right)$); ${R}_{v}$ is the turning radius of the UAV; and ${r}_{v}$ is the collision radius of the UAV.

In the ICS, the UAV receives repulsion vectors from the obstacles and an attraction vector from the target point, as follows:

12 $$\begin{array}{c}\left\{\begin{array}{c}{{\varvec{F}}}_{j}(P,V)={\left[\begin{array}{ccc}{F}_{j}^{x}& {F}_{j}^{y}& {F}_{j}^{z}\end{array}\right]}^{T}\\ {{\varvec{F}}}_{g}\left({\varvec{P}},{\varvec{V}}\right)={\left[\begin{array}{ccc}{F}_{g}^{x}& {F}_{g}^{y}& {F}_{g}^{z}\end{array}\right]}^{T},\end{array}\right.\end{array}$$

13 $$\begin{array}{c}\left|{{\varvec{F}}}_{j}\right|=\left\{\begin{array}{c}0,{d}_{j}>{R}_{j}\\ {\upvarepsilon }_{1},{d}_{j}\le {R}_{j},\end{array}\right.\end{array}$$

14 $$\begin{array}{c}\left|{{\varvec{F}}}_{g}\right|=\left\{\begin{array}{c}0, \exists \left|{{\varvec{F}}}_{j}\right|={\upvarepsilon }_{1}(j=1, 2,\dots )\\ 1, \forall \left|{{\varvec{F}}}_{j}\right|=0\left(j=1, 2,\dots \right),\end{array}\right.\end{array}$$

where *j* is the obstacle number (*j* = 1, 2 …); ${{\varvec{F}}}_{j}$ is the repulsive vector of the *j*th obstacle to the UAV, which points to the UAV from the obstacle, its size being determined by Eq. (13); ${d}_{j}$ is the distance between the UAV and the *j*th obstacle; and ${R}_{j}$ is the influence radius of the *j*th obstacle, ${\upvarepsilon }_{1}\gg 1$; ${{\varvec{F}}}_{g}$ is the attraction vector of the target point, which is directed from the UAV to the target point, its size being determined by Eq. (14).

Equations (13) and (14) show that after entering the radius of the obstacle, the UAV is prioritized to avoid the obstacle, no longer being affected by the attraction vector of the target point^48^. The total virtual vector received by the UAV can then be expressed as follows:

15 $$\begin{array}{c}{{\varvec{F}}}^{1}\left({\varvec{P}},{\varvec{V}}\right)={{\varvec{F}}}_{g}\left({\varvec{P}},{\varvec{V}}\right)+\sum_{j=1}^{n}{{\varvec{F}}}_{j}\left({\varvec{P}},{\varvec{V}}\right)={\left[\begin{array}{ccc}{F}_{x}& {F}_{y}& {F}_{z}\end{array}\right]}^{T}.\end{array}$$

Equation (15) obtains the virtual vector ${{\varvec{F}}}^{1}$ received by the UAV in the ICS, after which ${{\varvec{F}}}^{1}$ can be converted into a virtual vector under the VCS of the UAV, which can then be used to regulate the motion state of the UAV. The formula can be expressed as follows:

16 $$\begin{array}{c}{{\varvec{F}}}_{{\varvec{v}}}\left({\varvec{P}},{\varvec{V}}\right)={{\varvec{F}}}_{v}^{1}\left({\varvec{P}},{\varvec{V}}\right)={{\varvec{A}}}_{{\varvec{f}}{\varvec{v}}}\left({\varvec{P}}\right)\cdot {{\varvec{B}}}_{{\varvec{f}}{\varvec{v}}}\left({\varvec{P}}\right)\cdot {{\varvec{F}}}^{1}\left({\varvec{P}},{\varvec{V}}\right),\end{array}$$

17 $$\begin{array}{c}{{\varvec{A}}}_{{\varvec{f}}{\varvec{v}}}\left({\varvec{P}}\right)=\left[\begin{array}{ccc}\mathrm{cos}\theta & 0& \mathrm{sin}\theta \\ 0& 1& 0\\ -\mathrm{sin}\theta & 0& \mathrm{cos}\theta \end{array}\right],\end{array}$$

18 $$\begin{array}{c}{{\varvec{B}}}_{{\varvec{f}}{\varvec{v}}}\left({\varvec{P}}\right)=\left[\begin{array}{ccc}\mathrm{cos}\psi & \mathrm{sin}\psi & 0\\ -\mathrm{sin}\psi & \mathrm{cos}\psi & 0\\ 0& 0& 1\end{array}\right],\end{array}$$

where $${{\varvec{F}}}_{{\varvec{v}}}={\left[\begin{array}{ccc}{F}_{v}^{x}& {F}_{v}^{y}& {F}_{v}^{z}\end{array}\right]}^{T}$$ is the total virtual vector received by the UAV under the VCS; and ${{\varvec{A}}}_{{\varvec{f}}{\varvec{v}}}$ and ${{\varvec{B}}}_{{\varvec{f}}{\varvec{v}}}$ are the coordinate transformation matrices from the ICS to the VCS.

From this, it can be determined that the values of the velocity change trend matrix are as follows:

19 $$\begin{array}{c}\left\{\begin{array}{c}{f}_{l}={F}_{v}^{x}/\left|{F}_{v}^{x}\right|,{F}_{v}^{x}\ne 0\\ { f}_{\theta }={F}_{v}^{y}/\left|{F}_{v}^{y}\right|,{F}_{v}^{y}\ne 0\\ { f}_{\psi }={F}_{v}^{z}/\left|{F}_{v}^{z}\right|,{F}_{v}^{z}\ne 0\end{array}\right.{\text{o}}{\text{r}}\left\{\begin{array}{c}{f}_{l}=0,{F}_{v}^{x}=0\\ { f}_{\theta }=0,{F}_{v}^{y}=0\\ { f}_{\psi }=0,{F}_{v}^{z}=0.\end{array}\right.\end{array}$$

### Local minimum region separation method based on additional virtual vector

The traditional APF method has the problem that a UAV can easily fall into a local minimum in the process of path-solving. In this paper, the potential field function is expressed as a virtual vector, the path results obtained being based on the fixed-wing UAV model. Furthermore, the motion characteristics of a fixed-wing UAV that cannot hover are considered. Therefore, when the UAV enters a local minimum, it will not be unable to leave the region. However, there may be a situation in which the total repulsion vector from obstacles is parallel to the flight direction of the UAV. In this case, the UAV only receives a virtual vector with the opposite direction of the speed, and the UAV cannot take effective obstacle avoidance action. There needs to be a way to get the UAV out of such a scenario. At this point, the formation flight state of the UAV is $s={s}^{2}$—that is, the UAV is trapped in a local minimum region.

**Figure 3:**
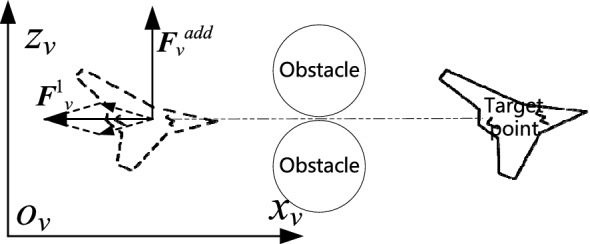
Schematic diagram of UAV, obstacle, and target point collinearity.

As shown in Fig. 3, under the VCS, using the ${o}_{v}{-x}_{v}-{z}_{v}$ plane as an example, the linear velocity direction of the UAV points to the target point, some obstacles appearing between the target point and the UAV. After the fixed-wing UAV enters the influence radius of the obstacle, it receives the virtual vector (${{\varvec{F}}}_{v}^{1}$) as shown in the figure. At this point, the UAV slows down but does not turn, so it cannot avoid the obstacle. When this situation is detected, the UAV can be given an additional virtual vector—such as ${{\varvec{F}}}_{v}^{add}$ ($\left|{{\varvec{F}}}_{v}^{add}\right|={\upvarepsilon }_{1}$)—which is in a positive direction along the ${o}_{v}-{z}_{v}$ axis, and the result of the planning algorithm indicates that the UAV will pull up while decelerating to avoid obstacles. Consequently, when the UAV enters a local minimum—that is, ${\varvec{S}}\in {{\varvec{S}}}^{2}$—the total virtual vector (${{\varvec{F}}}_{{\varvec{v}}}$) of the UAV under the VCS can be expressed as follows:

20 $$\begin{array}{c}{{\varvec{F}}}_{{\varvec{v}}}\left({\varvec{P}},{\varvec{V}}\right)={{\varvec{F}}}_{v}^{2}\left({\varvec{P}},{\varvec{V}}\right)={{\varvec{F}}}_{v}^{1}\left({\varvec{P}},{\varvec{V}}\right)+{{\varvec{F}}}_{v}^{add}\left({\varvec{V}}\right).\end{array}$$

### Formation forming and maintenance strategy based on speed dynamic adjustment

To better realize the formation flight of multiple UAVs, a formation forming and maintenance strategy based on speed dynamic adjustment is proposed by designing virtual vector dynamic change rules. During the formation forming stage, the wingmen accelerate to fly to a local path target point to create the formation. The lead plane is expected to slow down and await the wingmen for as long as all the wingmen have not yet reached the local path target point. At this stage, the formation flight state of the lead plane is ${s}_{0}={s}^{3}$. When all the wingmen are in formation, the lead plane then leads the formation, accelerating to the target area. At this stage, the formation members all enter the formation maintenance stage, their formation flight state being $s\in \left\{{s}^{1},{s}^{2}\right\}$. During this process, the formation may encounter obstacles, resulting in the destruction of their original formation, the process of formation forming and formation maintenance being repeated.

**Figure 4:**
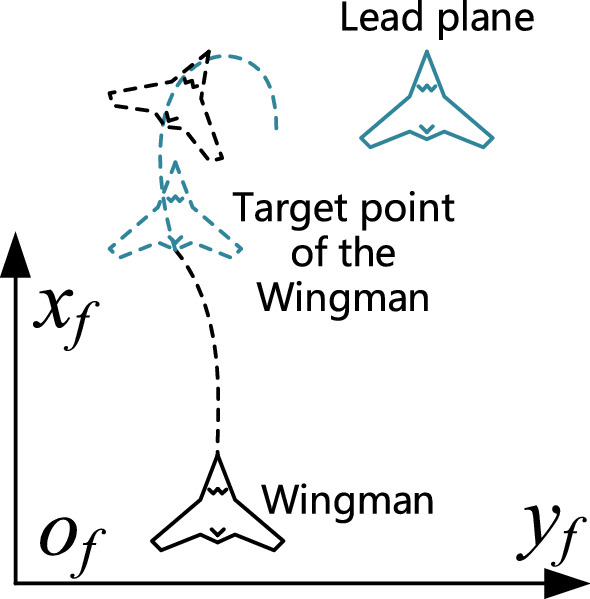
The scenario of the wingman arriving at a target point.

As shown in Fig. 4, the wingman—represented by the black solid line—reaches the local path target point—indicated by the blue dotted line—along the path of the black dotted line. However, at this stage, the lead plane—the solid blue line—is in a state of waiting. Consequently, the speed of the wingman is greater than that of the lead plane, causing the wingman to fly out of the accuracy range of the current target point. Due to the characteristics of the APF method and the UAV model, the wingman may take U-turn measures to return to the target point—shown by the blue dotted line in the figure—leading to formation instability. This situation could be addressed by slowing the wingman down ahead of time. Furthermore, when it reaches the effective range of the local path target point, it could maintain the same speed as the lead plane, which is similar to the “relay” problem.

However, the existence of obstacles can make it difficult for the wingman to accurately predict the flight trajectory and speed change law of the lead plane, making it difficult to accurately control its speed to match the motion of the lead plane when it reaches the local path target point. Consequently, the solution proposed in this study is to let the wingman estimate the distance from the target point and roughly determine the rate of deceleration required in advance to match the motion of the lead plane *i*(*i* > 0) when it reaches the ideal position. The formula for this process can be expressed as follows:

21 $$\begin{array}{c}\Delta {t}_{i}^{\boldsymbol{^{\prime}}}=({v}_{il}-{v}_{0l})/{a}_{il},\#\end{array}$$

22 $$\begin{array}{c}\Delta {d}_{i}^{\boldsymbol{^{\prime}}}={v}_{il}\Delta {t}_{i}^{\boldsymbol{^{\prime}}}-{a}_{il}({\Delta {t}_{i}^{\boldsymbol{^{\prime}}})}^{2}/2,\#\end{array}$$

23 $$\begin{array}{c}{f}_{il}=-1,{d}_{i}\le \Delta {d}_{i}^{\boldsymbol{^{\prime}}}.\#\end{array}$$

The linear velocities of the *i*th wingman and the lead plane at a certain time are ${v}_{il}$ and ${v}_{0l}$, respectively, and the linear acceleration of the wingman is ${a}_{il}$. The distance $\Delta {d}_{i}^{\boldsymbol{^{\prime}}}$ then required for the wingman to decelerate to the speed of the lead plane can be obtained from Eqs. (21) and (22). ${d}_{i}$ is the distance between the wingman and its local path target point. Then, Eq. (23) indicates that when $\Delta {d}_{i}^{\boldsymbol{^{\prime}}}$ is greater than ${d}_{i}$, the formation flight state of the wingman enters ${s}^{3}$, and the wingman must decelerate. After the wingman forms the formation, it leaves the ${s}^{3}$ state.

Based on the above rules, the total virtual vector (${{\varvec{F}}}_{{\varvec{v}}}$) received by the UAV under the VCS when the UAV is in the ${s}^{3}$ state can be expressed as follows:

24 $$\begin{array}{c}{{\varvec{F}}}_{v}\left({\varvec{P}},{\varvec{V}}\right)={{\varvec{F}}}_{v}^{3}\left({\varvec{P}},{\varvec{V}}\right)={\left[\begin{array}{ccc}{-\upvarepsilon }_{3}& 0& 0\end{array}\right]}^{T}+{{\varvec{F}}}_{v}^{s}\left({\varvec{P}},{\varvec{V}}\right),\#\end{array}$$

where $${\upvarepsilon }_{3}>0$$; $${{\varvec{F}}}_{v}^{s}={\left[\begin{array}{ccc}{F}_{v}^{sx}& {F}_{v}^{sy}& {F}_{v}^{sz}\end{array}\right]}^{T}\in \left\{{{\varvec{F}}}_{v}^{1},{{\varvec{F}}}_{v}^{2}\right\}$$; and $${\upvarepsilon }_{3}\gg |{F}_{v}^{sx}|$$.

The rationale for this method is that in an area with many obstacles, the primary task of each UAV is to avoid obstacles, making it difficult to create a formation. After the UAV leaves an area dense with obstacles, the lead plane can quickly adjust its attitude and fly to the target point in a stable state. At this stage, the wingman can accurately estimate the state of the lead plane and the distance from the local path target point.

### Simulation verification and analysis

The formation designed in this study consisted of five fixed-wing UAVs, including one lead plane and four wingmen. We stipulated the initial position, attitude, and performance parameters of each UAV and set the lead plane target point and formation requirements. A UAV was required to complete the formation flight in a preset environment. This paper stipulates that as long as the lead aircraft enters a certain range with the target point as the center, the path planning ends, and the wingman entering a certain range with its local path target point as the center can be regarded as the plane forming the formation. This paper calls it the target accuracy range. In this study, the time advance step of the algorithm *∆t* is 0.01 s. It also stipulates that all UAVs are isomorphic—that is, their performance is the same. The performance parameters are shown in Table 1.

**Table 1:** Performance parameters.

Parameter type	Value
UAVs'${R}_{o }(\mathrm{m})$	25
Initial ${v}_{l} \left(\mathrm{m}/\mathrm{s}\right)$	100
${a}_{l} (\mathrm{m}/{\mathrm{s}}^{2})$	10 (${f}_{l}>0$) 40 (${f}_{l}<0$)
${a}_{\theta } \left(\mathrm{rad}/\mathrm{s}\right)$	$\pi /6$
${a}_{\psi } \left(\mathrm{rad}/\mathrm{s}\right)$	$\pi /6$
Speed range	$300\ge {v}_{l}\ge 100 \left(\mathrm{m}/\mathrm{s}\right)$ $\pi /6\ge {v}_{\theta }\ge -\pi /6 \left(\mathrm{rad}/\mathrm{s}\right)$ $\pi /6\ge {v}_{\psi }\ge -\frac{\pi }{6}\left(\mathrm{rad}/\mathrm{s}\right)$
Target accuracy range ($$m)$$	10

### Comparative experiment of formation flight scene

The simulation experiments verified that the planning results of this study could meet the flight characteristics of fixed-wing UAVs and the requirements of formation forming and formation maintenance. Table 2 lists the initial parameters of the UAV formation members in the no-obstacle simulation scene, the target point of the lead plane being the target point in the ICS, and the local path target points of the wingmen being their positions in the VCS of the lead plane, determined by the specified formation. In this experimental scenario, the target formation is V-shaped.

**Table 2:** Initial parameters of the no-obstacle simulation scene.

UAV	Starting point $(x,y,z)/\mathrm{m}$	Attitude $(\theta ,\psi )/\mathrm{rad}$	Target point $(x,y,z)/\mathrm{m}$
Lead plane 0 (blue)	(1500, 2000, 300)	(0, 0)	(6000, 7000, 4800)
Wingman 1 (yellow)	(2000, 1500, 0)	(0, − $\pi$)	(− 300, 300, 0)
Wingman 2 (red)	(1500, 1500, 200)	(0, $- \pi$)	(− 600, 600, 0)
Wingman 3 (green)	(1500, 3000, 300)	(0, $- \pi /2$)	(− 300, − 300, 0)
Wingman 4 (black)	(1500, 4000, 300)	(0, $- \pi /2$)	(− 600, − 600, 0)

**Figure 5:**
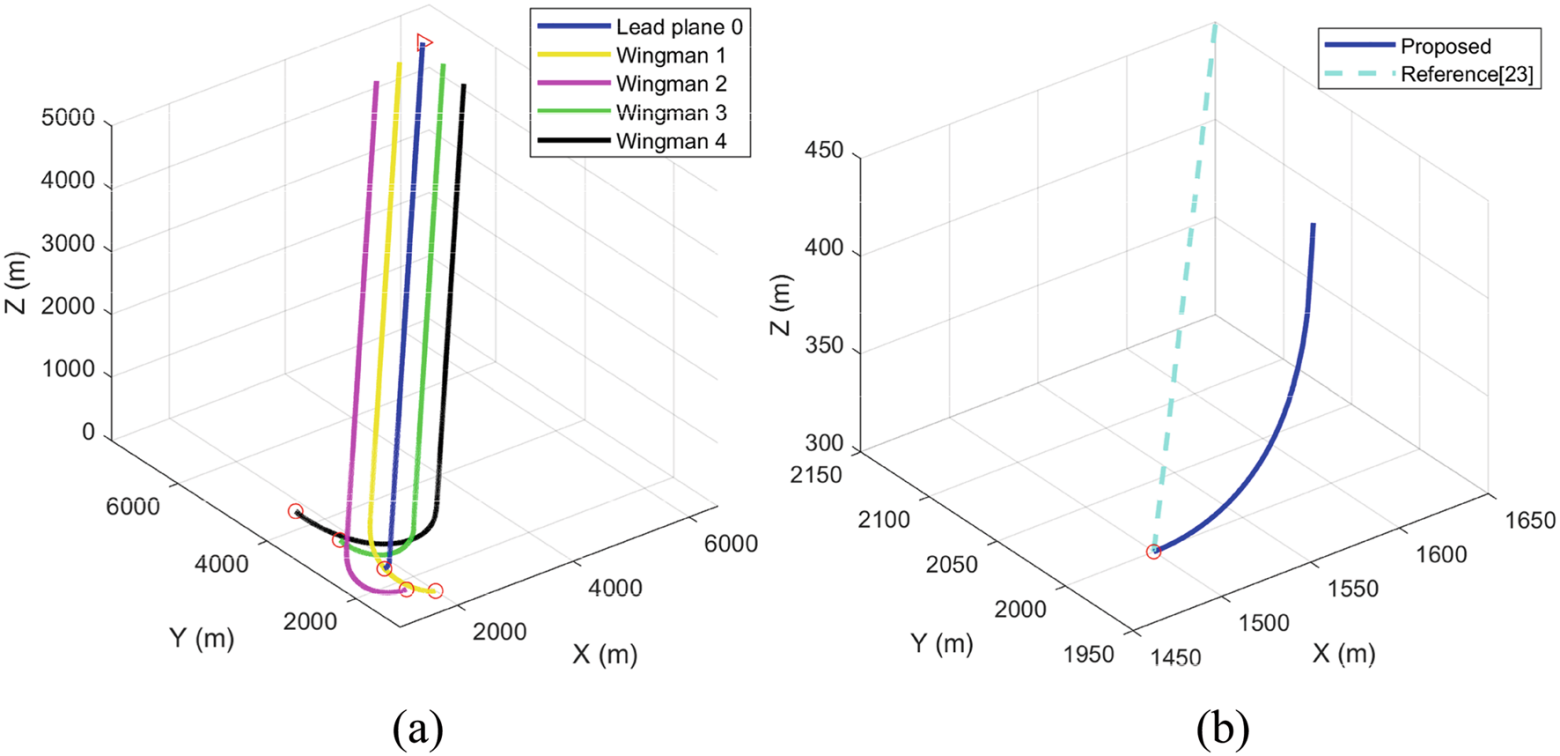
Flight trajectory planning results of the UAV formation flight (**a**) The whole path of the formation; (**b**) The path of the lead plane near the starting point.

The path planning method of UAV formation based on the PPF method proposed in this paper was used to plan the mission scenarios specified in Table 2. As shown in Fig. 5a, the red circle represents the starting point of each UAV, and the red triangle represents the target point of the lead plane. As can be seen from the figure, owing to the highest priority, the lead plane (blue curve) does not consider the influence of the wingmen's position on itself and flies to the target point, while the wingmen calculate and follow the local path target point based on the lead plane’s position and preset formation parameters and avoid the friendly plane based on their priority.

The APF-based method proposed in Ref.^23^ is a classical method to solve the path planning of UAV formation. Using this method to plan this scenario, the local view of the starting point of the lead plane is shown in Fig. 5b. As shown by the light blue dotted line in this figure, the planned path of the lead plane is a straight line connecting the starting point and the endpoint, as this method ignores the turning angle constraints of fixed-wing UAVs. The PPF-based method proposed in this study solves this problem. As shown by the dark blue solid line in Fig. 5b, the lead plane adjusts the direction of flight based on its initial direction.

**Figure 6:**
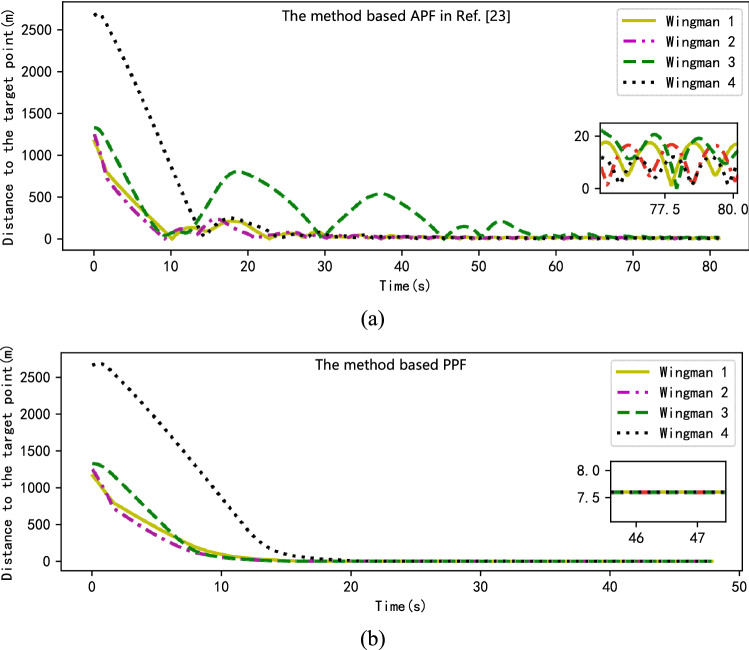
The situation of wingmen following the target points (**a**) Method proposed in Ref.^23^; (**b**) method proposed in this paper.

Figure 6 shows the situation of wingmen following their local path target point in the planning results. As shown in Fig. 6a, the results obtained using the APF-based method vibrate considerably when the UAVs are forming formation, their trajectories unable to stabilize during the later stages of planning—as shown in the enlarged inset of Fig. 6a. By contrast, the track obtained using the PPF-based method is smoother, the trajectory stabilizing during the formation flight stage after the formation process.

It can be seen from Fig. 6b that all the wingmen reach the local path target point at 22 s and enter the formation maintenance stage. As can be seen from the enlarged inset of Fig. 6b, although there is still a certain distance between the wingmen and their local path target points during the formation maintenance stage, these distances are all within 10 m—that is, the UAVs are located in a 10 m sphere of radius with the target points as the center—which meets the target accuracy range summarized in Table 1.

**Figure 7:**
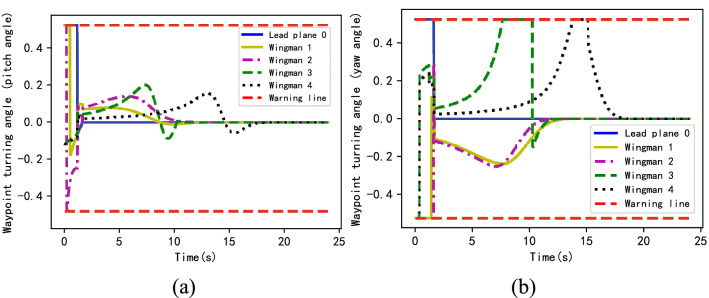
The waypoint turning angle (**a**): pitch angle; (**b**): yaw angle.

Figure 7 shows the situation of pitch and yaw angles changing the corresponding track points of each formation member during the first 25 s of the planning result. Based on the time step (*∆t*) and the angular velocity range (Table 1), the turning angle threshold of the track point can be obtained as shown by the red dotted line in the figure, the thresholds being $\pm \pi /6$ rad, respectively. It can be seen from the figure that the turning angles of the results planned using the method in this paper are all limited to the prescribed range, which meets the kinematic constraints of the UAV, due to the small step (0.01 s). The turning angle in the figure is multiplied by a gain coefficient (*K*) to facilitate the display, in which K = 100/rad.

**Figure 8:**
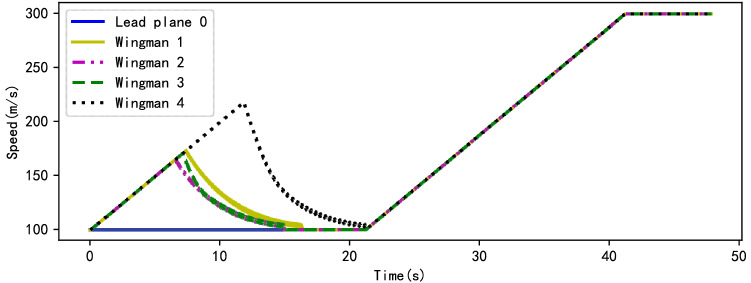
Speed variation curve of UAVs.

In the process of formation flight, the planning algorithm will adjust the speed of each UAV according to its current state, so as to get a smooth and stable trajectory. Figure 8 shows the velocity curve of each UAV. As shown in the figure, in the initial stage, the leader plane keeps flying at a low speed, and the wingmen accelerate toward the leader plane. When the wingmen are about to form a formation, they begin to slow down gradually. After all the wingmen reach the position specified by the formation, the leader plane begin to lead all the members to accelerate towards the target area. Thus it can be seen that the speed change of UAV is consistent with the result of path planning.

**Figure 9:**
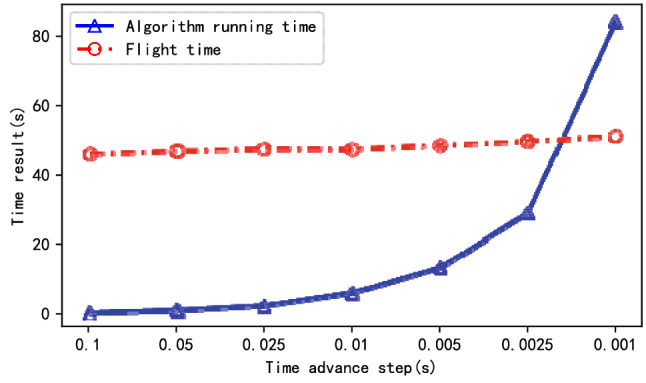
The execution time of the PPF-based algorithm and the flight time of the UAV formation in the simulation under different ***∆t.***

Due to the characteristics of real-time solution of the online planning method, the planning method proposed in this paper can output the results of the current moment and calculate the results of the next step at the same time, which is different from the global planning method to output the complete result after the solution is completed. And this is one of the reasons why online algorithms can deal with dynamic environments. In the above experimental scenario, the PPF-based planning algorithm is run according to different time advance step *∆t*, and the comparison between the running time of the algorithm and the flight time of the planning result is shown in Fig. 9.

As can be seen from the figure, the running time of the algorithm is directly related to the time advance step *∆t*. The running speed of the algorithm will decrease with the decrease of *∆t* (the improvement of planning accuracy), and its running time will even exceed the flight time of the UAV specified in the planning results. Therefore, when using this method in dynamic environment, users need to consider the relationship between efficiency and accuracy, so as to improve the accuracy of planning results as much as possible under the premise of real-time requirements (that is, the algorithm outputs the results while the UAVs follow the results).

### Simulation verification and analysis of complex scenes

The simulation experiments in the scene with obstacles verified the cooperative flight ability of the formation in a complex environment, including the ability to avoid obstacles and follow the target point. To reflect the reliability of our proposed method, this section modified the experimental scene based on Table 2, where the obstacles simulated restricted areas such as a no-fly zone in the actual flight scene. The modified parts are summarized in Table 3.

**Table 3:** Initial parameters of the simulation scene with obstacles.

UAV	Target point $(x,y,z)/\mathrm{m}$	Obstacle	Location $(x,y,z,{R}_{o})/\mathrm{m}$
Lead plane 0 (blue)	(6000, 7000, 300)	Obstacle 0	(3000, 3000, 100, 350)
Wingman 1 (yellow)	(− 100, 100, 0)	Obstacle 1	(3500, 4500, 100, 650)
Wingman 2 (red)	(− 200, 200, 0)	Obstacle 2	(5000, 4500, 100, 650)
Wingman 3 (green)	(− 100, − 100, 0)	Obstacle 3	(4500, 6000, 100, 850)
Wingman 4 (black)	(− 200, − 200, 0)		

**Figure 10:**
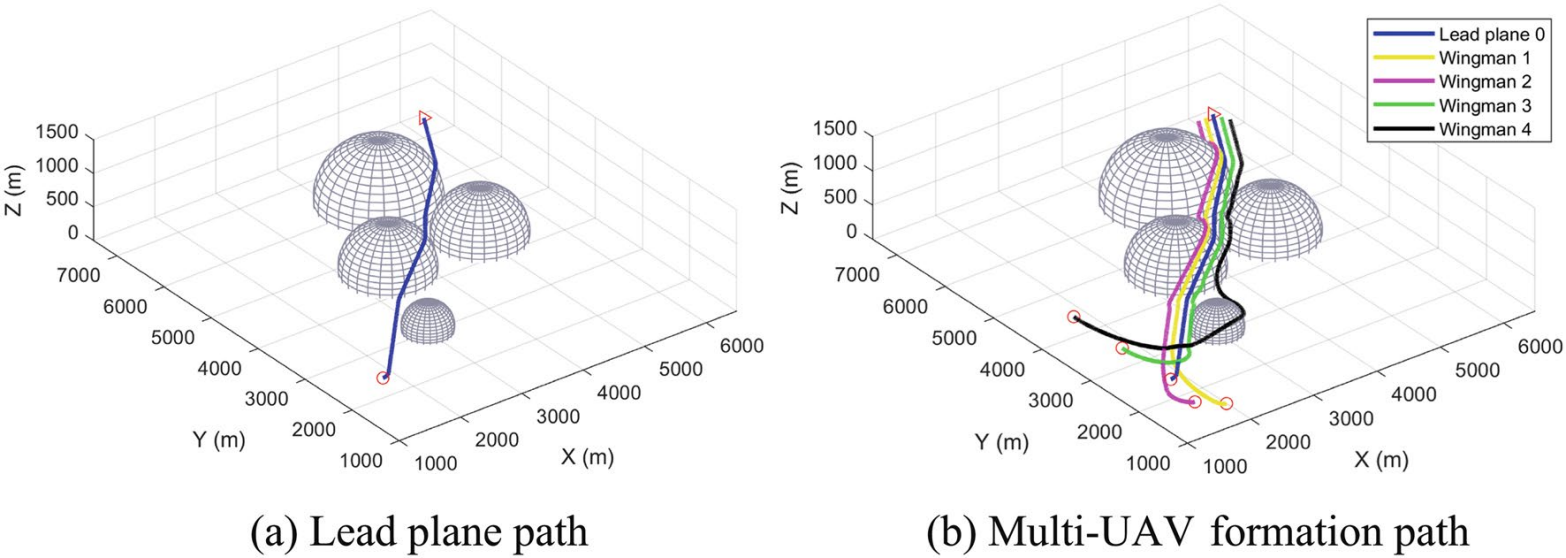
Multi-UAV path planning in a space with obstacles.

First, we verified the path-planning ability of a single UAV in a scene with obstacles. The method proposed in this study was applied to the scene, the result of UAV path planning in a complex three-dimensional space as shown in Fig. 10a.

As shown in Fig. 10a, the lead plane starts from the starting point—that is, the red circle. During the flight, the lead plane encounters obstacles and takes measures to avoid them. After leaving an obstacle, the lead plane immediately adjusts its flight direction to the target point—that is, the red triangle. Finally, the method proposed in this study plans a smooth path from the starting point to the endpoint of the lead plane.

Based on the starting points set in Table 2, the fixed-wing UAV formation uses the tighter formation set in Table 2 to follow the lead plane to the target area. The results of planning using our method are shown in Fig. 10b. During the initial stage, the wingmen fly to their respective local path target points. At some point, the formation enters the obstacle area. Using Wingman 4 as an example, we can see from this figure that the local path target point of Wingman 4 coincides with Obstacle 0. Consequently, based on the principle of prioritizing obstacle avoidance, Wingman 4 temporarily leaves the formation and adopts the obstacle avoidance operation. After bypassing Obstacle 0, Wingman 4 immediately resumes the tracking formation.

**Figure 11:**
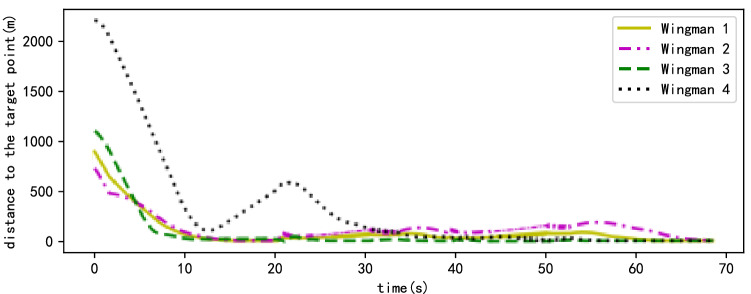
The situation of wingmen following the local path target points in the scene with obstacles.

Figure 11 shows that the wingmen repeatedly switch between the formation forming and formation maintenance stages owing to the complexity of the simulation scene until they leave the area dense with obstacles. However, each wingman follows their local path target points as much as possible, the distances between the wingmen and their target point being kept to a minimum. The results of path planning show the flight time of the planned path to be 68.5 s. Owing to the limitations of complex scenes, UAV formation is not fully formed in most cases. However, the UAVs still rapidly enter the overall formation maintenance stage from 68.29 s.

**Figure 12:**
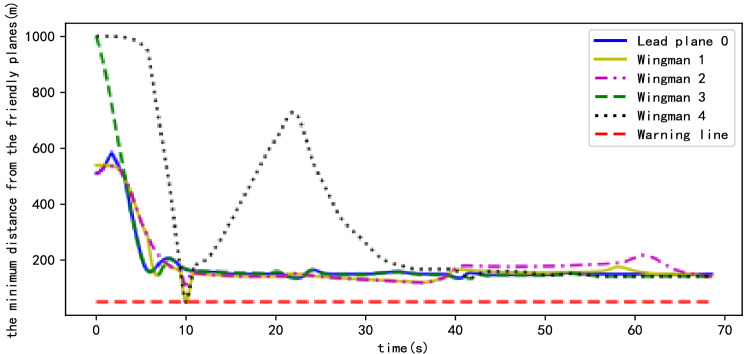
Minimum distance between formation members and friendly aircraft in the scene with obstacles.

In a complex environment, flying in a fixed formation is no longer the focus of planning, with coordinated flight and mutual avoidance between friendly UAVs being more important. Figure 12 shows the minimum distance between each UAV and its friends during the formation flight. Among them, Wingman 4 stays away from other friendly planes for some time because of it prioritizing obstacle avoidance, which is consistent with the situation shown in Fig. 10b. At approximately 10 s, the distance between Wingman 1 and Wingman 4 changes considerably, but because of the avoidance rule, the latter successfully avoids the former, the distance between them always being more than the safe distance—that is, the red dotted line in Fig. 12, the safe distance being 50 m. At the same time, each wingman quickly follows the target point after obstacle avoidance, the distance between the wingmen and the target point being maintained at a minimum, demonstrating the effectiveness of the cooperative flight method proposed in this study.

**Figure 13:**
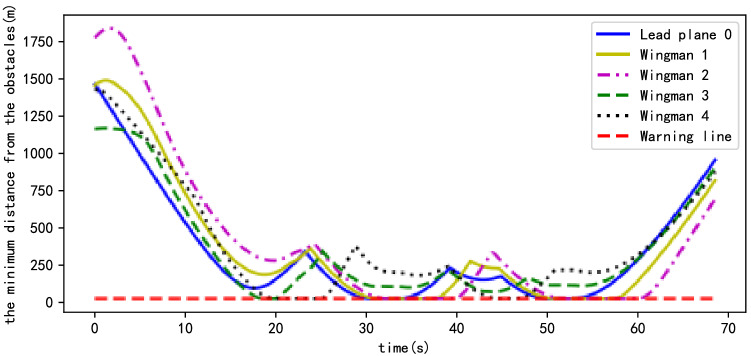
Minimum distance between UAVs and obstacles.

In an environment dense with obstacles, the UAV's avoidance of obstacles is also important. Figure 13 shows the minimum distance from each UAV to the surface of the obstacles. Because the UAVs can navigate obstacles while flying in formation, it can be seen that the trends of corresponding curves of each UAV are similar. Moreover, when the UAVs navigate the obstacle, they immediately enter the formation assembly phase, Fig. 10b showing that several UAVs fly along the surface of the obstacle, which is consistent with the curves shown in Fig. 13—for example, that of Wingman 2. However, owing to the existence of obstacle avoidance rules, the minimum distance between each UAV and the surface of the obstacle is above the safe distance—that is, the red dotted line in Fig. 13, the safe distance being 25 m.

## Conclusions

In this study, a kinematic model suitable for fixed-wing UAVs was established based on the motion characteristics of a fixed-wing aircraft. Based on the “lead-plane–wingman” formation structure, a teammate avoidance strategy was set and the local path target points of the wingmen in the process of formation flight were determined using the lead plane as the center. The formation flight state of the UAV was divided into three states, and a path planning method based on PPF was proposed to realize trajectory generation in different states.

Firstly, the potential field function was represented by a virtual vector. The attraction vector from the target point and the repulsion vector from the obstacle were set, and the obstacle avoidance rules were designed, before a formation trajectory that satisfied the kinematic constraints was obtained based on the UAV and its formation model. Secondly, the local minimum region where the UAV, obstacle, and target points were collinear was detected, and an additional potential field vector perpendicular to the current course was set to break away from this region. Finally, by designing the dynamic change rule of the potential field vector, a formation and maintenance strategy based on speed dynamic adjustment was proposed. The lead plane was required to slow down and wait for the wingmen during the formation stage. The wingmen estimated the motion state of the lead plane, adjusting their own motion state, so as to ensure the stability of the planned trajectory in the formation maintenance phase and meet the requirements of path smoothing. Through simulations of a formation flight scene, it was verified that this method could realize the formation flight of multiple UAVs, and unlike the APF-based method, the proposed path planning method could consider the UAV turning constraints, resulting in a smoother path. Through the simulations of complex scenes with fixed obstacles, the effectiveness of the coordination and obstacle avoidance measures among UAVs during multi-UAV formation flight was verified, and the track planned using this method was shown to be conflict-free.

In future work, we will further study the optimization of path-planning results, the impact of irregular obstacles on UAV formation, countermeasures under abnormal conditions (such as lead plane failure) and the influence and solution of random factors such as communication delay and positioning error on UAV path tracking in the actual flight environment.

## Data Availability

The datasets generated during and/or analysed during the current study are available from the corresponding author on reasonable request.

## References

[1] Wei X, Xu J (2021). Distributed path planning of unmanned aerial vehicle communication chain based on dual decomposition. Wirel. Commun. Mob. Comput..

[2] Qadir Z, Ullah F, Munawar HS, Al-Turjman F (2021). Addressing disasters in smart cities through Uavs path planning and 5G communications: A systematic review. Comput. Commun..

[3] Sivakumar, M. & Naga Malleswari, T. Y. J. A literature survey of unmanned aerial vehicle usage for civil applications. *J. Aerosp. Technol. Manag.* **13**, (2021).

[4] Rajasree, R. & Jisha, V. R. Optimal formation control of unmanned aerial vehicles with reconfiguration. In *2015 International Conference on Control, Communication & Computing* 36–41 (IEEE, 2015).

[5] Wang Y, Yue Y, Mao S, He L, Wang D (2021). Formation reconstruction and trajectory replanning for multi-Uav patrol. IEEE/ASME Trans. Mechatron..

[6] Faridi AQ, Sharma S, Shukla A, Tiwari R, Dhar J (2018). Multi-robot multi-target dynamic path planning using artificial bee colony and evolutionary programming in unknown environment. Intell. Serv. Robot..

[7] Seiler, P., Pant, A. & Hedrick, K. *Analysis of Bird Formations* 118–123 (IEEE, 2002).

[8] Sharma, A., Shoval, S., Sharma, A. & Pandey, J. K. Path planning for multiple targets interception by the swarm of Uavs based on swarm intelligence algorithms: A review. In *Technical Review—IETE*. 1–23 (2021).

[9] Zhang H, Xin B, Dou L, Chen J, Hirota K (2020). A review of cooperative path planning of an unmanned aerial vehicle group. Front. Inform. Technol. Electron. Eng..

[10] Aggarwal S, Kumar N (2020). Path planning techniques for unmanned aerial vehicles: A review, solutions, and challenges. Comput. Commun..

[11] Choi K, Kim J (2021). Uav path planning method for avoiding restricted areas. Intell. Serv. Robot..

[12] Liu Q (2021). Multi-Uav path planning based on fusion of sparrow search algorithm and improved bioinspired neural network. IEEE Access..

[13] Naderi, K., Rajamäki, J. & Hämäläinen, P. *Rt-Rrt: A Real-Time Path Planning Algorithm Based on Rrt*. 113–118 (ACM, 2015).

[14] Tian, J., Wang, Y. & Yuan, D. *An Unmanned Aerial Vehicle Path Planning Method Based on the Elastic Rope Algorithm*. 137–141 (IEEE, 2019).

[15] Liu G, Shu C, Liang Z, Peng B, Cheng L (2021). A modified sparrow search algorithm with application in 3D route planning for Uav. Sensors..

[16] Tao RW, Wen T, Chen H (2011). A real-time 3D motion planning and simulation scheme for nonholonomic systems. Simul. Model. Pract. Theory..

[17] Phung MD, Ha QP (2021). Safety-enhanced Uav path planning with spherical vector-based particle swarm optimization. Appl. Soft. Comput..

[18] Liang X, Meng G, Xu Y, Luo H (2018). A geometrical path planning method for unmanned aerial vehicle in 2D/3D complex environment. Intell. Serv. Robot..

[19] Feng J (2021). Uav dynamic path planning based on obstacle position prediction in an unknown environment. IEEE Access..

[20] Goerzen C, Kong Z, Mettler B (2010). A survey of motion planning algorithms from the perspective of autonomous Uav guidance. J. Intell. Rob. Syst..

[21] Sun J, Tang J, Lao S (2017). Collision avoidance for cooperative Uavs with optimized artificial potential field algorithm. IEEE Access..

[22] Saravanakumar S, Asokan T (2013). Multipoint potential field method for path planning of autonomous underwater vehicles in 3D space. Intell. Serv. Robot..

[23] Chen Y, Yu J, Su X, Luo G (2015). Path planning for multi-Uav formation. J. Intell. Robot. Syst..

[24] Jothi A, Priyadarsini LKP (2022). Optimal path planning for intelligent Uavs using graph convolution networks. Intell. Autom. Soft Comput..

[25] Liu, K., Xv, C., Huang, D. & Ye, X. *Uav Path Planning Based on Improved Whale Optimization Algorithm*. 569–573 (IEEE, 2021).

[26] Roberge V, Tarbouchi M, Labonte G (2013). Comparison of parallel genetic algorithm and particle swarm optimization for real-time Uav path planning. IEEE Trans. Ind. Inform..

[27] Huan, L., Ning, Z. & Qiang, L. *Uav Path Planning Based on an Improved Ant Colony Algorithm*. 357–360 (IEEE, 2021).

[28] Qiannan, Z., Ziyang, Z., Chen, G. & Ruyi, D. *Path Planning of Uavs Formation Based on Improved Ant Colony Optimization Algorithm*. (Yantai, 2014).

[29] Chen, J., Ye, F. & Jiang, T. *Path Planning Under Obstacle-Avoidance Constraints Based on Ant Colony Optimization Algorithm*. 1434–1438 (IEEE, 2017).

[30] Chen Q (2018). Path planning for Uavs formation reconfiguration based on dubins trajectory. J. Cent. S. Univ..

[31] Gul F, Mir I, Abualigah L, Sumari P, Forestiero A (2021). A consolidated review of path planning and optimization techniques: Technical perspectives and future directions. Electronics.

[32] Duan H, Yu Y, Zhang X, Shan S (2010). Three-dimension path planning for Ucav using hybrid meta-heuristic Aco-De algorithm. Simul. Model. Pract. Theory.

[33] Jamshidi V, Nekoukar V, Refan MH (2021). Real time Uav path planning by parallel grey wolf optimization with align coefficient on can bus. Clust. Comput..

[34] Zhou Y, Su Y, Xie A, Kong L (2021). A newly bio-inspired path planning algorithm for autonomous obstacle avoidance of Uav. Chin. J. Aeronaut..

[35] Selvam, P. K., Raja, G., Rajagopal, V., Dev, K. & Knorr, S. Collision-free path planning for Uavs using efficient artificial potential field algorithm. In *2021 IEEE 93rd Vehicular Technology Conference (VTC2021-Spring)*, 1–5 (2021).

[36] Liu, Z., Wang, X. & Li, K. Research on path planning of multi-rotor Uav based on improved artificial potential field method. In *MATEC Web Conferences*, 7006 (2021).

[37] Wang B, Bao J, Zhang L, Sheng Q (2018). Uav autonomous path optimization simulation based on radar tracking prediction. Eurasip J. Wirel. Commun. Netw..

[38] Bai X (2021). Uav path planning based on improved a∗ and Dwa algorithms. Int. J. Aerosp. Eng..

[39] Muslimov TZ, Munasypov RA (2020). Adaptive decentralized flocking control of multi-Uav circular formations based on vector fields and backstepping. Isa Trans..

[40] Manathara, J. G. & Ghose, D. Rendezvous of multiple Uavs with collision avoidance using consensus. *Int. J. Aerosp. Eng.* (2012).

[41] Paul, T., Krogstad, T. R. & Gravdahl, J. T. Uav formation flight using 3D potential field. In *2008 Mediterranean Conference on Control Automation*. (2008).

[42] Feng, Y., Wu, Y., Cao, H. & Sun, J. Uav formation and obstacle avoidance based on improved Apf. In *10th International Conference on Modelling, Identification and Control (ICMIC)*, 1–6 (2018).

[43] Zhao, Y., Jiao, L., Zhou, R. & Zhang, J. Uav formation control with obstacle avoidance using improved artificial potential fields. In *36th Chinese Control Conference* (2017).

[44] Zhang, J., Yan, J., Zhang, P. & Kong, X. Collision avoidance in fixed-wing Uav formation flight based on a consensus control algorithm. *IEEE Access*. 43672–43682 (2018).

[45] Zhang J, Yan J, Zhang P (2018). Fixed-wing Uav formation control design with collision avoidance based on an improved artificial potential field. IEEE Access..

[46] Liu W, Zheng X, Deng Z (2021). Dynamic collision avoidance for cooperative fixed-wing Uav swarm based on normalized artificial potential field optimization. J. Cent. S. Univ..

[47] Fang, Y., Yao, Y., Zhu, F. & Chen, K. Fixed-wing Uav kinematics model using direction restriction for formation cooperative flight. In *12th International Conference on Simulation and Modeling Methodologies, Technologies and Applications*, 92–101 (Science and Technology Publications, 2022).

[48] Vasarhelyi, G. *et al*. Optimized flocking of autonomous drones in confined environments. *Sci. Robot.* **3**, (2018). 10.1126/scirobotics.aat3536 33141727

